# Decomposition Dynamics and Changes in Chemical Composition of Wheat Straw Residue under Anaerobic and Aerobic Conditions

**DOI:** 10.1371/journal.pone.0158172

**Published:** 2016-07-05

**Authors:** Hongjian Gao, Xi Chen, Junling Wei, Yajie Zhang, Ligan Zhang, Jiang Chang, Michael L. Thompson

**Affiliations:** 1 School of Resources and Environment, Anhui Agricultural University, Hefei 230036, People’s Republic of China; 2 State Key Lab of Tea Plant Biology and Utilization, Anhui Agricultural University, Hefei 230036, People’s Republic of China; 3 Agronomy Department, Iowa State University, Ames, Iowa 50011, United States of America; Old Dominion Univ., UNITED STATES

## Abstract

Soil aeration is a crucial factor that regulates crop residue decomposition, and the chemical composition of decomposing crop residues may change the forms and availability of soil nutrients, such as N and P. However, to date, differences in the chemical composition of crop straw residues after incorporation into soil and during its decomposition under anaerobic vs. aerobic conditions have not been well documented. The objective of the present study was to assess changes in the C-containing functional groups of wheat straw residue during its decomposition in anaerobic and aerobic environments. A 12-month incubation experiment was carried out to investigate the temporal variations of mass, carbon, and nitrogen loss, as well as changes in the chemical composition of wheat (*Triticum aestivum* L) straw residues under anaerobic and aerobic conditions by measuring C-containing functional groups using solid state nuclear magnetic resonance (NMR) spectroscopy. The residual mass, carbon content, and nitrogen content of the straw residue sharply declined during the initial 3 months, and then slowly decreased during the last incubation period from 3 to 12 months. The decomposition rate constant (*k*) for mass loss under aerobic conditions (0.022 d^-1^) was higher than that under anaerobic conditions (0.014 d^-1^). The residual mass percentage of cellulose and hemicellulose in the wheat straw gradually declined, whereas that of lignin gradually increased during the entire 12-month incubation period. The NMR spectra of C-containing functional groups in the decomposing straw under both aerobic and anaerobic conditions were similar at the beginning of the incubation as well as at 1 month, 6 months, and 12 months. The main alterations in C-containing functional groups during the decomposition of wheat straw were a decrease in the relative abundances of O-alkyl C and an increase in the relative abundances of alkyl C, aromatic C and COO/N-C = O functional groups. The NMR signals of alkyl C and aromatic C in decomposing wheat straw residues under anaerobic condition were higher than those under aerobic conditions. The higher mass percentages of lignin and the higher signals of aromatic C and alkyl C functional groups in decomposing wheat residues under anaerobic conditions than under aerobic conditions were due to the slower decomposition rates of aryl C and alkyl C in wheat straw residues under anaerobic conditions.

## Introduction

Approximately 3.8 billion tons of crop residues are produced annually in the world [1]. Crop residues can be returned to the soil for nutrient recycling, and they are an important source of organic matter to improve soil physical, chemical and biological properties [2, 3]. In addition to organic carbon, crop residues contain around 3.0 to 8.2 kg of nitrogen, 0.2 to 0.6 kg of phosphorous, and 7.2 to 23.3 kg of potassium per ton dry matter [4]. Returning crop residues to the soil is widely recognized as a useful approach to recycle nutrients, increase soil fertility, and prevent impoverishment of organic carbon in the soil [4]. Crop residues contain significant quantities of organically bound nutrients, such as N and P, which may not be readily available for subsequent crop use because they must first undergo decomposition processes [5, 6]. The decomposition of crop residue is governed by both quantity and quality of the residue [7–10], climatic conditions such as temperature and moisture [11], and soil properties [12]. Crop residue quality, particularly nitrogen, lignin, and polyphenol concentrations, may alter decomposition dynamics [8, 13–15]. Low-quality plant residues with high carbon/nitrogen ratios, lignin, and other aromatic compounds decompose more slowly than high-quality plant litters [16]. Soil water content is also an important factor that affects the decomposition of plant litter [17], first because soil water potential must be low enough to provide water for microbial activity. In addition, significant differences in the oxidation-reduction potential and soluble oxygen concentration between anaerobic and aerobic conditions lead to differences in the activity of soil microbial communities [18–20]. Some studies have indicated that the decomposition of rice straw proceeded more slowly under anaerobic conditions than under aerobic conditions [21]. In anaerobic environments, due to a lack of free oxygen that is required for its de-polymerization, lignin exhibits a greater resistance to degradation than other plant constituents [20]. The accumulation of partially degraded lignin residues in soil organic matter (SOM) of an anaerobic soil has been shown to be greater than that of a comparable aerobic soil [22]. Humification of SOM was also influenced by soil aerobic and anaerobic conditions, soil aeration appeared to promote SOM humification due to the accumulation of unsubstituted and alkyl-substituted aromatic C [22]. The immobilization of soil N by SOM and mineralization of N from SOM are also impacted by soil aeration conditions [23]. However, to date, many details about plant litter decomposition in anaerobic and aerobic environments remain unclear.

The wheat-rice rotation is a very popular agricultural practice in the Yangtze and Huai River region, China. Rice is generally grown as a wetland crop, so the soil is usually flooded during the cropping season and drained after harvesting. Wheat is generally grown as a dryland crop, and soil is usually aerated during the cropping season. Therefore, during the rice-wheat rotation, soils cycle through aerobic and anaerobic conditions throughout the year. In these regions, rice is planted quickly after wheat is harvested in May or June, and wheat is planted quickly after rice is harvested in September or October. Thus decomposition of wheat or rice straw in a rice-wheat rotation field occurs after it has been incorporated into soil and may occur under either aerated or flooded conditions [24].

Our previous studies found that the incorporation of wheat residues led to an initial period of N deficiency and a decline of rice yields compared with the treatments with the same dose of chemical fertilizer application alone [24]. Some reasons for this phenomenon include (1) microbes may consume crop residues as an energy source and compete with crop plants for available N [25], (2) incorporation of crop residues may increase the losses of N through volatilization of NH_3_, N denitrification, or leaching [4, 24], and (3) decomposed crop residues may contain chemical compounds or functional groups that can make soil N less plant-available [25]. The increased N loss and competition of available N between microbes and crop plants after returning crop residue to soil have been well documented [25, 4], but the impacts of chemical composition of crop residues on N forms and availability are still not clear. We postulated that aeration conditions play a dominant role in the decomposition of wheat straw and therefore the chemical structure of decomposed wheat straw residues. If so, the concentrations of C-containing functional groups in decomposing wheat residues may alter depending on the anaerobic and aerobic conditions, and thus change the forms and availability of soil nutrients, such as N and P. However, differences in the chemical composition of wheat straw residues after incorporation into soil and during its decomposition under anaerobic *vs*. aerobic conditions have not been well documented.

The objective of the present study was to assess changes in the chemical composition of wheat straw during its decomposition in anaerobic and aerobic environments. The temporal variations of mass, carbon, and nitrogen in wheat straw were also investigated during a 12-month incubation. We also documented changes in the contents of cellulose, hemicelluloses and lignin as well as relative abundance of C-containing functional groups during wheat straw decomposition.

## Materials and Methods

### Study Site

Wheat (*Triticum aestivum L*) straw decomposition trials were conducted at the agriculture experimental station of Anhui Agricultural University (AAU) in Hefei, China (E116°41; N31°30). This area experiences a typical central-subtropical climate. The average annual precipitation is 985 mm, and the annual average temperature is 15.5°C. The annual frost free days are 227, and the annual sunshine duration is 2100 hours.

### Experimental design

#### Soil and wheat straw sampling and preparation

The soil at the research site is classified as a Hapli-Udic Cambisol in the Chinese Soil Taxonomy (corresponding to an Inceptisol in the US Soil Taxonomy) [26], with pH of 6.2, organic matter concentration of 11.4 g kg^-1^, and total nitrogen concentration of 0.52 g kg^-1^. Soil alkali-hydrolyzable (1.0 mol L^-1^ NaOH) nitrogen concentration is 41.5 mg kg^-1^, Bray 1 (mixed solution of 0.03 mol L^-1^ NH_4_F and 0.025 mol L^-1^HCl) extractable phosphorous concentration is 6.4 mg kg^-1^, and 1.0 mol L^-1^ ammonium acetate-extractable potassium concentration is 121.8 mg kg^-1^. The contents of clay, silt and sand were 452, 383 and 165 g kg^-1^, respectively [27].

Wheat straw was collected after grain harvest, cut into 1 cm pieces, and dried at 50°C to a constant weight prior to further analysis. Concentrations of carbon and nitrogen in the wheat straw were determined by an Elementar Vario EL cube elemental analyzer through dry combustion at 900°C [28]. Concentrations of phosphorous and potassium in wheat straw were analyzed by Mo-Sb colorimetry and flame photometry after acidic digestion by concentrated H_2_SO_4_ (98%) and H_2_O_2_ (30%) [27]. Organic carbon, total nitrogen, total phosphorus, and total potassium concentrations in the wheat straw before experiment were 479.8 mg kg^-1^, 8.8 mg kg^-1^, 2.2 mg kg^-1^, and 9.8 mg kg^-1^, respectively.

#### Decomposition trial

Six grams of wheat (*Triticum aestivum L*) straw samples were put into a double-layer nylon mesh bag that was 20 cm long and 20 cm wide. The mesh size of the litter bags was 0.15 mm, which could separate the wheat straw from soil aggregates and mesofauna outside the bag yet allow soil water and microorganisms access to the wheat straw in the litter bag. The plots (in triplicate) simulating anaerobic and aerobic conditions were established according to a completely random design in the field. Six plots (3 m long × 4 m wide) constructed with 1-cm thick waterproof polyvinyl fluoride panels were placed in the wheat-corn rotation field by vertical insertion into soil, 50 cm of the panel was buried in the soil and 10 cm of the panel was left above ground. Within each plot, bags were inserted into the soil at about 20-cm depth and equidistantly distributed. To ensure adequate soil contact, all the sides of litter bags were filled in soil and completely packed, a 3-5-cm layer of soil was also placed over the bags. Soil in three plots was flooded and kept with a 1-cm water layer above soil surface during the entire incubation period, simulating anaerobic condition. Soil in the other frames was kept at 75%~80% of the maximum water-holding capacity by irrigation in the entire incubation period, simulating aerobic conditions. In situ soil moisture was detected by Time domain Reflectometry with Intelligent MicroElements (TRIME-PICO 32 TDR, Germany). The oxidation-reduction (redox) potentials were measured every five days by insertion of an oxidation-reduction potential sensor into the soil. Normally, the soil redox potential was determined at 4 PM, except during bad weather such as rain or snow. The measured oxidation-reduction potentials remained at about -100 ± -50 mv and 200 ± 50 mv in the anaerobic and aerobic treatments, respectively. There were no wheat or rice plants growing in the plots during the entire 12-month incubation period.

The mass of each litter bag and wheat straw was separately weighed before incubation. The wheat straw sample was then placed in each bag and incubated under the simulated anaerobic and aerobic conditions for 0, 0.5, 1, 3, 6 and 12 months. At the end of each incubation period, three bags of wheat straw residues were sampled from each anaerobic and aerobic treatment plot, respectively. The mass of each litter bag and wheat straw residue was recorded, and the dry mass of the residue was determined after removal from the nylon bags and drying at 50°C. The dried samples were ground to pass through a 0.15-mm mesh for chemical property analysis and then further ground into a powder for NMR analysis.

#### Chemical analysis

The contents of cellulose, hemicellulose and lignin in wheat straw were determined using a modification of the method proposed by Van Soest and his collaborator [29, 30]. Briefly, the first step of the sequential extraction was the removal of the neutral detergent extractable fraction (NDF) at 100°C for 60 min, which left the cell wall fraction composed of primarily of hemicellulose, cellulose, and lignin. The second step was an acid detergent extraction using 2 M HCl, which removed hemicellulose. This was followed by a strong acid (H_2_SO_4_, 72%) extraction, which removed cellulose. The non-extractable residue was assumed to be mainly composed of lignin and insoluble proteins. All solid fractions obtained after each extraction were oven dried at 80°C, and the residual organic matter was estimated by loss on ignition at 550°C for 4 h. The results were calculated as percentages of the volatile solids. The mass percentages of cellulose, hemicellulose and lignin in wheat straw before the trial were 38.1%, 25.4%, and 10.1%, respectively.

#### Nuclear magnetic resonance (NMR) spectroscopy analysis

^13^C NMR spectroscopy analyses were performed using a Bruker Avance III 400 spectrometer at 100 MHz (400 MHz ^1^H frequency). All experiments were run in a double resonance probe head using 4-mm sample rotors. The ^13^C multiple ramped amplitude cross polarization/magic angle spinning (^13^C multiCP) NMR experiments were performed. Straw samples were packed into 4-mm rotors and a 2-mm height glass insert was put at the bottom of rotor to keep soil samples within the radio frequency coil. The spectra were recorded at a spinning speed of 14 kHz, with a recycle delay of 0.5s. The 90° pulse-lengths were 4.2 μs for ^1^H, and 4 μs for ^13^C[31]. The ^13^C NMR signals in the ^13^C multiCP spectra were assigned to different functional groups following [16, 32]. After the multiCP analysis, multiCP combined with recoupled dipolar dephasing experiments were applied to generate a subspectrum with nonprotonated carbons such as COO/N−C = O, nonprotonated aromatics, OC_q_O, and mobile groups such as mobile OCH_3_, CCH_3_, and mobile (CH_2_)_n_ groups [33].

#### Carbon-13 cross polarization/total sideband suppression with and without dipolar dephasing

Qualitative composition information was obtained with good sensitivity by ^13^C cross polarization/total sideband suppression (CP/TOSS). The NMR experiments were conducted at a spinning speed of 5 kHz and a CP time of 1 ms, with a ^1^H 90° pulse length of 4 μs and a recycle delay of 1 s. Four-pulse total suppression of sidebands [32] was used before detection, and two-pulse phase-modulated decoupling was applied for optimum resolution. The corresponding subspectrum with signals of nonprotonated carbons and carbons of mobile groups such as rotating CH_3_ was obtained by ^13^C CP/TOSS combined with 40-μs dipolar dephasing. The relative abundances of different carbon functional groups were obtained by integrating signal intensities with various chemical shift regions, and they are reported as the percentage of the total signal region from 0 to 220 ppm. The CP/TOSS NMR spectra are generally assigned to eight dominant carbon groups [33–36]; they are: (1) alkyl C (0–44 ppm); (2) Methoxyl/N-alkyl (44–64 ppm); (3) O-alkyl C (64–93 ppm); (4) anomeric C (93–113 ppm); (5) aromatic C (113–142 ppm); (6) aromatic C-O (142–164 ppm); (7) carboxyl/amide C (164–188 ppm); and (8) ketone/aldehyde C (188–220 ppm).

#### Carbon-13 chemical shift anisotropy (CSA) filter

To separate the signals of anomeric carbons (O–C–O) from those of aromatic carbons, both of which may resonate approximately between 90 and 120 ppm, the aromatic-C signals were selectively suppressed by a five-pulse ^13^C CSA filter with a CSA-filter time of 35 μs [36, 37]. To select the signals of nonprotonated O–C–O (ketal) carbons, which may extend to 120 ppm, this CSA filter was combined with a dipolar dephasing time of 40 μs. In a complementary experiment, selected spectra of protonated anomerics (O–CH–O, acetals) were obtained by CSA filtering after short CP. The details of this technique have been described elsewhere [36, 37].

#### Spectral editing of immobile CH_2_ and CH

The combined spectrum of these chemical groups was obtained with good sensitivity in a simple spectral-editing experiment. First, a CP/TOSS spectrum was recorded using a short CP of 40 μs. It showed predominantly protonated carbons in immobile segments, but residual peaks of quaternary carbons resulted from two-bond magnetization transfer. Second, a CP/TOSS spectrum was acquired using a short CP of 40-μs dipolar dephasing. It contained only the residual signals of quaternary carbons or mobile segments (including CH_3_ groups with >50% efficiency). This residual spectrum was then subtracted from the first CP/TOSS spectrum. The resulting difference spectrum represents immobile CH_2_ and CH carbons, with a small CH_3_ contribution [37]. The details of this technique have been described elsewhere [37].

#### Data analysis

The decomposition of wheat straw was expressed in percentage of mass, carbon and nitrogen loss in wheat straw residues from the original materials. The decomposition process was simulated using a modification of the model proposed by Berg and Ekbohm [38, 39] and Berg et al.[40]:

$$y=y_{0}\left(1\;-a\cdot e^{-kt}\right)$$ (1)

where *y* is the fraction of the initial mass, C or N remaining at time *t* (months), *k* the decomposition rate constant (d^-1^), *a* is the fraction of the initial mass, C, or N of the material that is subject to loss, and *y*_*0*_ is the asymptote value. The half-lives of the C or N lost from wheat straw residues were calculated using the formula: *t*_1/2_ = ln2/*k*_i_ = 0.693/*k*_i_, where *k*_i_ is the decomposing rate constant of C or N fraction [9].

Statistical analyses were performed using the Statistical Package for Social Science (SPSS 18.0). Analysis of variance and least significant difference tests were performed to determine the statistical significance (*p* = 0.05) of the mass loss and C and N released from wheat straw residues at different incubation periods under anaerobic and aerobic conditions.

## Results

### Time-dependent mass loss from wheat straw

**Fig 1:**
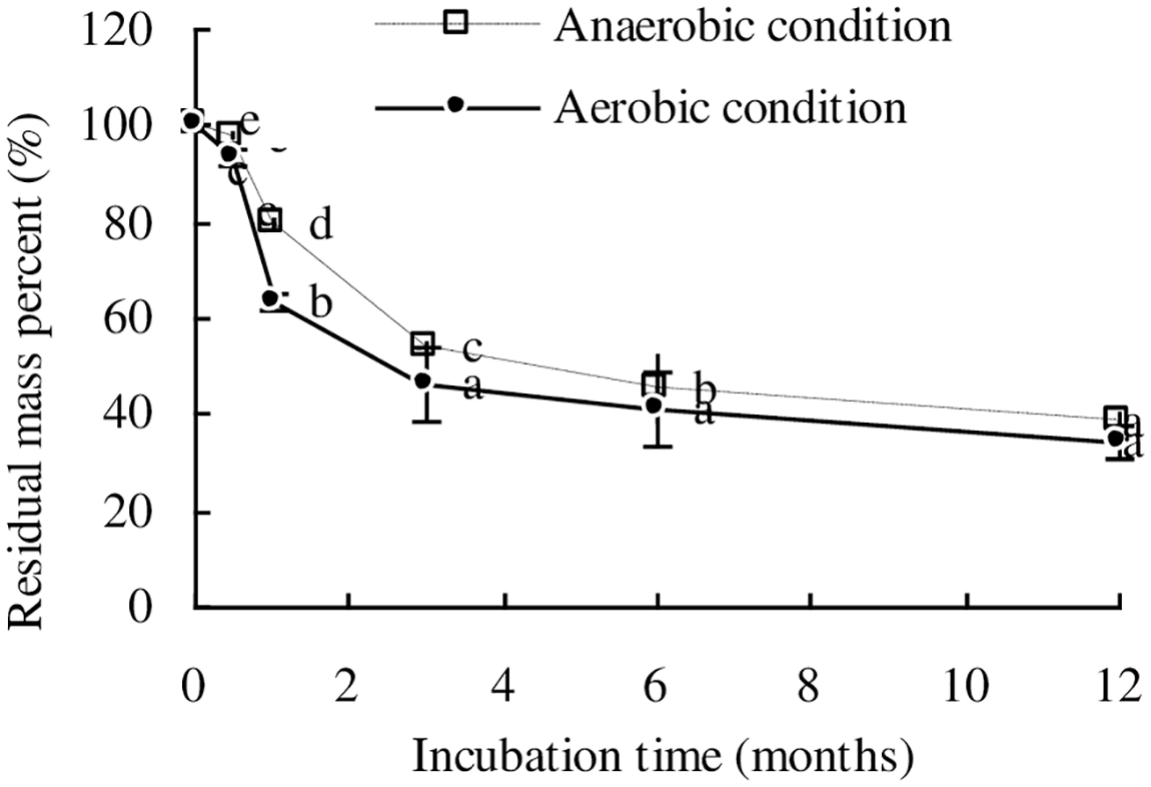
Temporal variation of the residual mass percent of wheat straw.

During the incubation period, the residual mass of wheat straw sharply decreased in the first 3 months and then slowly decreased from 3 to 12 months under both the anaerobic and aerobic conditions (Fig 1). The losses of wheat straw mass in the first 3 months were 46.2% and 53.7% of its initial mass under anaerobic and aerobic conditions, respectively. Wheat straw mass loss in the anaerobic condition was 3.8% to 16.1% slower than that in the aerobic condition during the entire incubation period. During the first month, the cumulative mass loss of wheat straw in the aerobic condition was 36.6% of the initial mass, which was significantly higher (*p*<0.05) than that in the anaerobic condition (20.5%). This difference in mass loss between the anaerobic and aerobic conditions was less during the incubation period from 3 to 12 months, with a difference of only 4.2% between the two treatments at 12 months.

The decomposition rate constants (*k*) of wheat straw in the anaerobic and aerobic conditions were 0.014 *d*^-1^ and 0.022 *d*^-1^, respectively, and the half lives (*t*_1/2_) of wheat straw decomposition in the anaerobic and aerobic condition were 122.2 d and 72.8 d, respectively (Table 1). These results indicated that wheat straw decomposition in the anaerobic condition was slower than in the aerobic condition.

**Table 1:** Regression models of the wheat residual mass and incubation time.

Treatments	*y* = *y*_*0*_(1−*a·e*^*-kt*^)			
	*y*_*0*_	*a*	*k*	R^2^
Aerobic condition	36.71	66.17	0.022	0.96
Anaerobic condition	38.55	65.2	0.014	0.98

Note: *y* is the percentage of the initial mass remaining at time *t* (months); *y*_0_ is the asymptotic value when time is ∞, *a* is the percentage of the initial mass of material subject to loss; *k* is the decomposition rate constant calculated by the least-squares method of fitting the model (*d*^-1^); *t* is the incubation time (months); and R^2^ is a correlation coefficient.

### Time-dependent carbon and nitrogen loss from wheat straw

**Fig 2:**
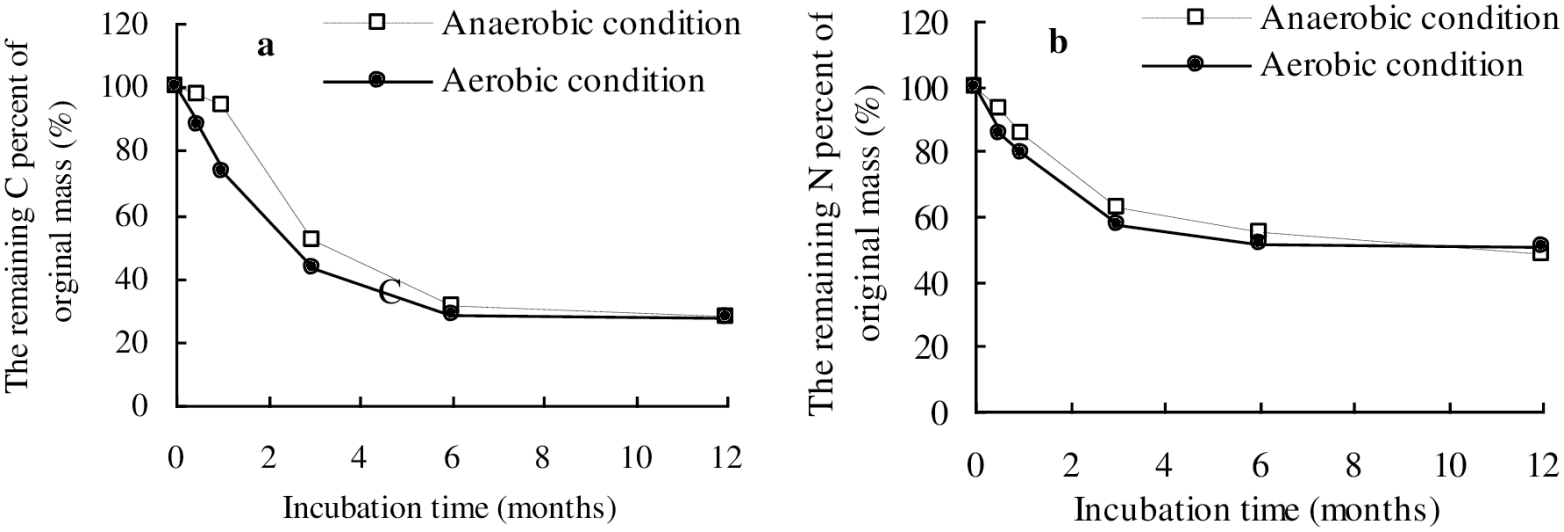
Temporal variation of the remaining C (a) and N (b) as a percentage of the original mass in decomposing wheat straw.

The loss of carbon from wheat straw was higher from 0 to 6 months than that from 6 to 12 months. Carbon lost from wheat straw in the first 6 months accounted for 69.9% and 71.4% of the original carbon mass in the anaerobic and aerobic condition (Fig 2a), respectively. About 73% of carbon was lost from the wheat straw in both the anaerobic and aerobic conditions during the 12-month incubation. The carbon loss rate constants (*k*) were 0.0152 *d*^-1^ and 0.0085 *d*^-1^, and the half lives (*t*_1/2_) of carbon lost from wheat straw were 114.8 d and 72.8 d under anaerobic and aerobic conditions, respectively. This revealed that carbon loss from wheat straw under aerobic conditions was faster than that under anaerobic conditions.

The loss of nitrogen from wheat straw in the first 3 months was higher than that in the later 9 months (Fig 2b). Nitrogen lost from wheat straw during the first 3 months of incubation accounted for 37.8% and 42.4% of the original nitrogen mass in the anaerobic and aerobic condition, respectively. The nitrogen loss rate constant (*k*) under anaerobic conditions (0.012 *d*^-1^) was lower than that under aerobic conditions (0.02 *d*^-1^) (Table 2).

**Table 2:** Regression models of carbon and nitrogen lost from wheat straw.

Treatments	Carbon lost / *C*_t_ = *C*_0_(1−*e*^-*kCt*^)			Nitrogen lost / *N*_t_ = *N*_0_(1−*e*^-*kNt*^)		
	*C*_0_	*k*_C_	R^2^	*N*_0_	*k*_N_	R^2^
Aerobic condition	2.15	0.0152	0.99	26.4	0.02	0.995
Anaerobic condition	2.31	0.0085	0.95	27.7	0.012	0.993

Note: *C*_t_ is organic carbon lost from wheat straw at time *t* (g); *C*_0_ is the initial potentially mineralizable carbon (g); *k*_c_ is the carbon decomposition rate constant (*d*^-1^); R^2^ is the correlation coefficient; *N*_t_ is nitrogen lost at time *t* (mg); *N*_0_ is the initial potentially mineralizable nitrogen (mg); *k*_N_ is the nitrogen decomposition rate constant calculated (*d*^-1^); R^2^ is the correlation coefficient.

### Time-dependent changes of cellulose, hemicelluloses and lignin in wheat straw

**Fig 3:**
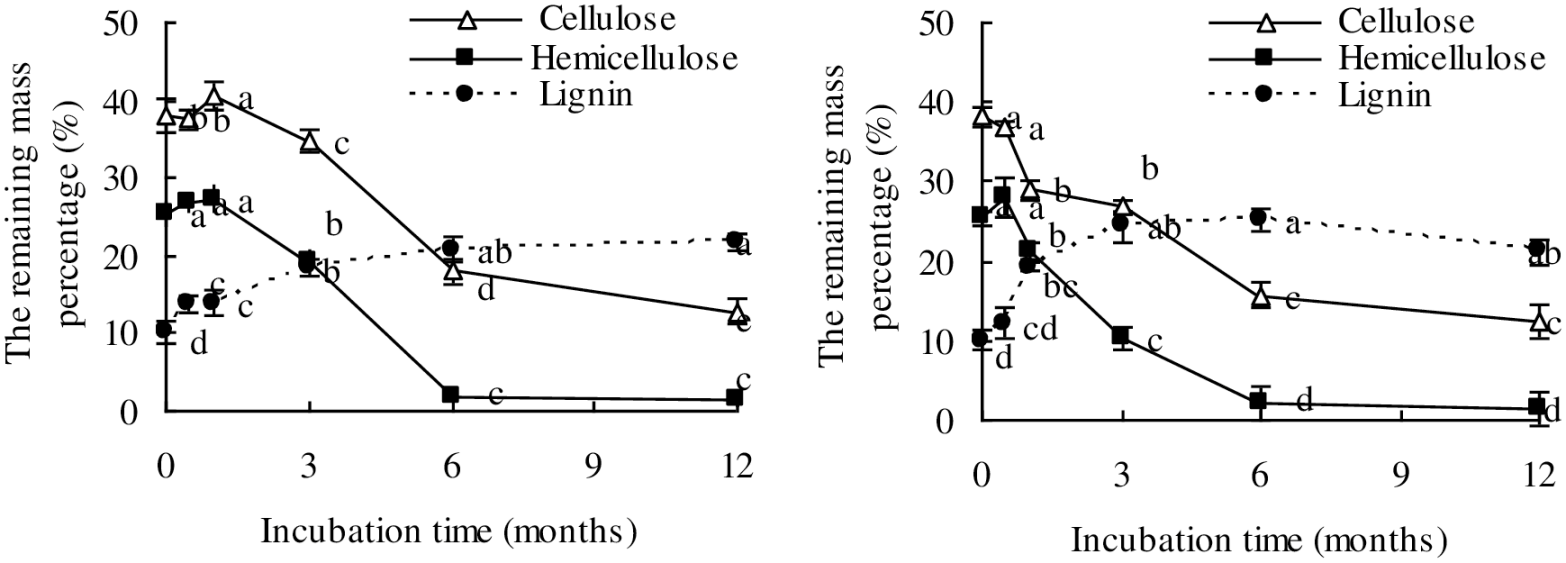
Temporal variations in the remaining mass percentage (%) of cellulose, hemicellulose, and lignin in wheat straw residues under anaerobic (A) and aerobic (B) conditions. Notes: Error bars indicate the standard deviation of the triplicate samples.

The mass loss of cellulose and hemicellulose in wheat straw sharply declined from 0 to 6 months, and then it slowly declined from 6 months to 12 months under both anaerobic and aerobic conditions (Fig 3). The remaining mass percentage of cellulose in wheat straw was higher than that of hemicellulose under both anaerobic and aerobic conditions. The remaining mass percentage of lignin in wheat straw increased gradually from 10.1% to 21.8% during the entire incubation period under the anaerobic conditions, whereas under the aerobic conditions it increased from 10.1% to 25.1% during the first 6 months, and then declined slightly in the 12^th^ month.

### NMR Spectroscopy

**Fig 4:**
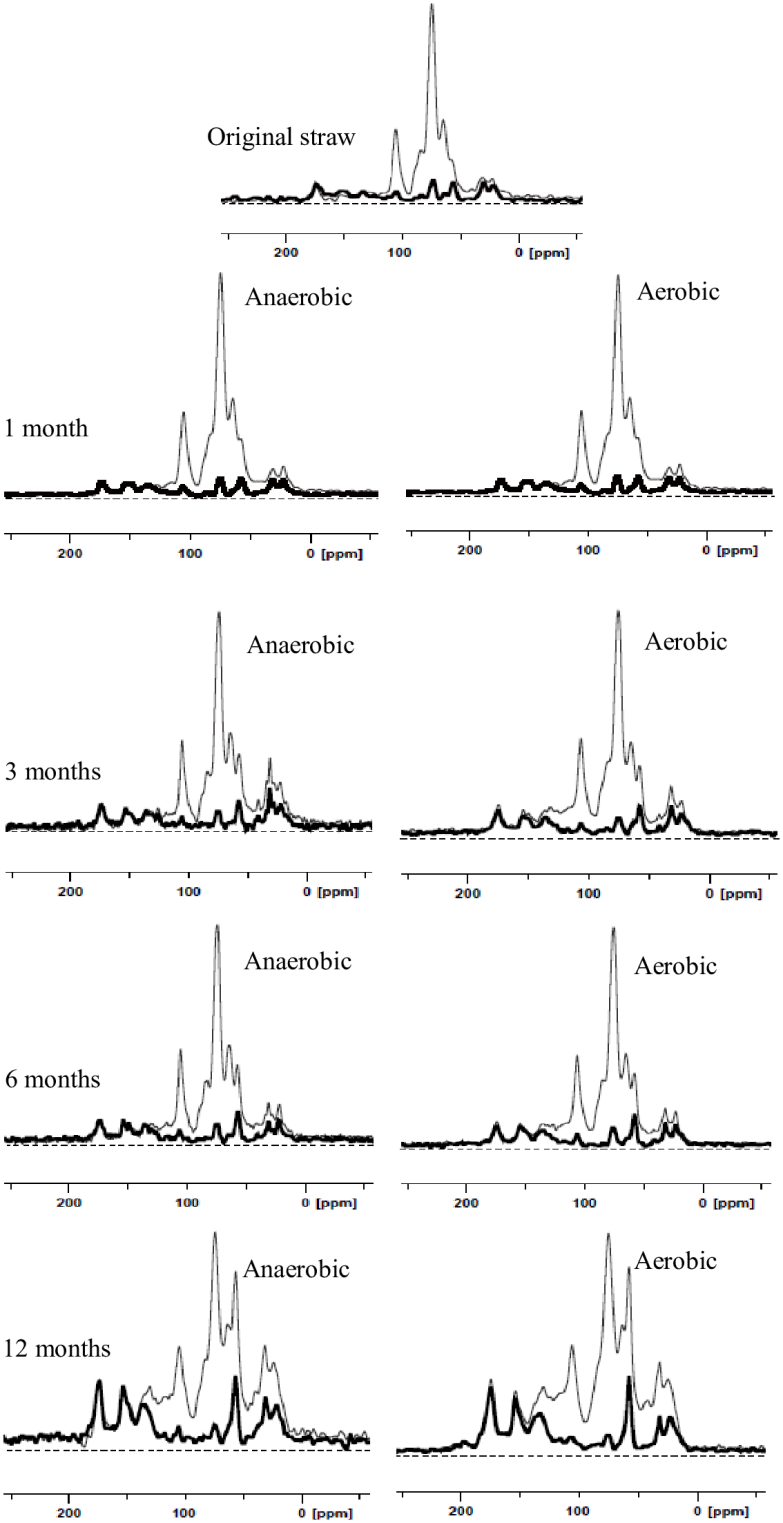
^13^C multiCP NMR spectra (thin lines) and multiCP/DD NMR spectra (thick lines) of original and decomposing wheat straw at different incubation stages.

The ^13^C multiCP NMR spectra of the original and decomposed wheat straw samples under anaerobic and aerobic conditions are displayed in Fig 4. The ^13^C multiCP spectra (thin lines) show signals from all carbon sites, while multiCP with dipolar dephasing (multiCP/DD) spectra (thick lines) highlight signals from nonprotonated carbons and mobile carbons including COO/N−C = O, aromatic C–O, nonprotonated aromatics, nonprotonated quaternary carbon (OCqO), OCH_3_, CCH_3_ and mobile (CH_2_)*n* groups.

The spectra of C-containing functional groups were similar during the decomposition of wheat straw from 0 to 12 months under both anaerobic and aerobic conditions (Fig 4). The alkyl C (0–44 ppm) region showed signals from CH_2_ groups, such as those from long-chain polymethylene structures (e.g., fatty acids, waxes, and biopolyesters) and terminal methyl groups from both alkyl compounds and acetyl substituents in plant hemicellulose [41]. In the region of 44–64 ppm, the bands assigned to the methoxyl C (O-CH_3_) and N-alkyl groups (NCH) were likely derived from guaiacyl and syringyl lignin components, and/or C-N bonds in amino acids or peptides, respectively [35]. The signals of O-alkyl C (64–93ppm) represented the overlapping resonances of carbons in the pyranoside structure of cellulose and hemicellulose [41]. The peak of anomeric C (93–113 ppm) was associated with the anomeric C-1 carbon of cellulose and hemicellulose present in plant material [35]. The broad band around (113–142 ppm) was attributed to the presence of lignin-derived aromatic C [42, 43].

The small peak in the region 142–162 ppm was attributed to O-aryl C, and the aromatic C-O indicated the presence of lignin and tannin components. Finally, the sharp signal at 172 ppm was due to carboxyl groups (in aliphatic acids of plant and microbial origins) and/or amide groups in amino acid moieties [44, 45]. The corresponding ^13^C multiCP spectra after 40-μs dipolar dephasing (Fig 4 thick line) show only the signals from nonprotonated carbons and carbons of mobile functional groups including COO/N-C = O, aromatic C-O, nonprotonated aromatics, OCqO, OCH_3_, CCH_3_, and mobile (CH_2_)_n_ groups [32]. In agreement with the similarities of their multiCP NMR spectra, the dipolar dephasing spectra of the initial and decomposing wheat straw for both aerobic and anaerobic conditions share many common features, e.g., the signals of CCH_3_ around 22 ppm, slightly mobile CCH_2_C around 30 ppm, OCH_3_ between 50 and 60 ppm, nonprotonated aromatic C-C around 130 ppm, aromatic C-O around 150 ppm and COO/N-C = O around 173 ppm [32]. The multiCP and multiCP/DD spectra showed the contribution of the signal from both OCH_3_ and NCH at 57 ppm. The resonances at 20 ppm (CH_3_) and 173 ppm (COO/N-C = O) reflected the presence of CH_3_COO in hemicellulose and a possible small contribution of the N-acetyl in peptide residues [36]. The signals at 57 and 173 ppm were partially associated with proteins and peptides. Signals from OCH_3_ and aromatic C-O carbons reflected the presence of lignin. Signals in the unsaturated carbon region between 113 and 142 were attributed to aromatic carbon in lignin or olefinic carbons in lipids [45].

**Fig 5:**
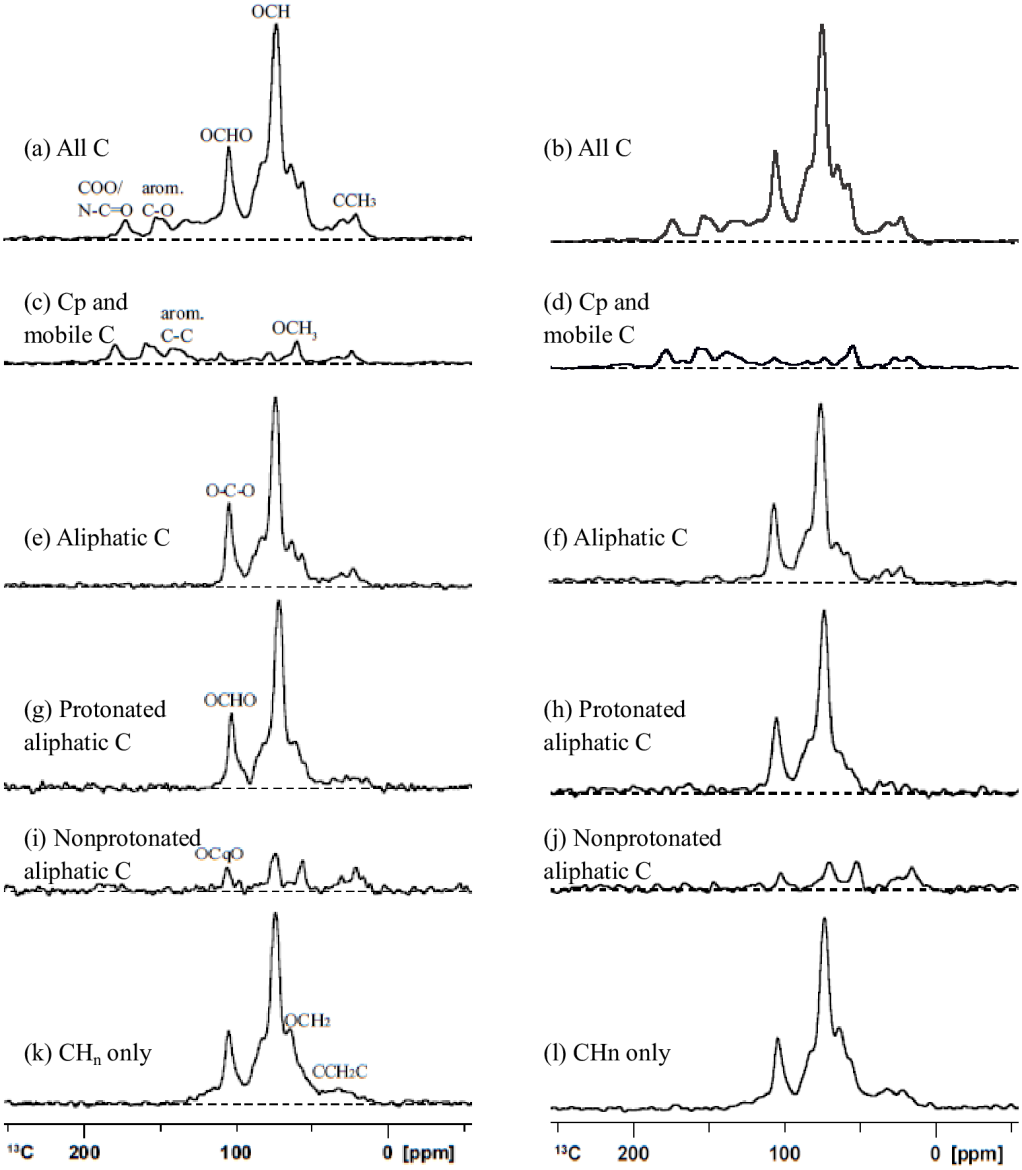
Spectral editing for identification of functional groups in 3-month decomposing wheat straw under anaerobic (left) and aerobic (right) conditions: (a, b) nonselective cross polarization/total sideband suppression (CP/TOSS) spectra for reference, with a contact time of 1 ms at a spinning speed of 5 kHz; (c, d) corresponding dipolar dephasing CP/TOSS spectra showing nonprotonated carbons and mobile carbons, acquired after 40-μs of decoupling gated off; (e, f) selection of *sp*^3^-hybridized carbon signals by a ^13^C chemical shift anisotropy (CSA) filter with 35-μs filter time, other parameters as in (a); (g, h) corresponding selection of protonated *sp*^3^-hybridized C signals with a 35-μs CSA filter and short 50-μs CP; (i, j) selection of nonprotonated or mobile *sp*^3^-hybridized C signals with a 35-μs CSA filter and 40-μs dipolar dephasing; (k, l) selection of relatively immobile CH and CH_2_ signals with small residual CH_3_, which was achieved by finding the difference between a short-CP spectrum and a spectrum of short CP combined with dipolar dephasing. All the spectra were normalized to the highest peak. Recycle delays for all the spectra were 1 s and the number of scans was 6144.

The differences of C-containing functional groups in decomposing wheat residues from 0 to 3 months were relatively higher than those from 3 to 12 months under both anaerobic and aerobic conditions. We selected the 3^rd^ month as the critical time for further NMR spectral editing. Fig 5a and 5b show the nonselective cross polarization/total sideband suppression (CP/TOSS) spectra displaying different C-containing functional groups in 3-month decomposed wheat straw under anaerobic (left) and aerobic (right) conditions, respectively. Fig 5c and 5d illustrate the corresponding dipolar dephasing CP/TOSS spectra showing nonprotonated carbons and mobile carbons. The ^13^C CSA filtered spectra are displayed in Fig 5e and 5f, those with ^13^C CSA and short CP are depicted in Fig 5g and 5h, and the spectra with CSA and dipolar dephasing are shown in Fig 5i and 5j. All the samples contained considerable anomeric carbons, whose signals resonated around 105 ppm and are distinguishable in the ^13^C CSA-filtered spectra, demonstrating the existence of linked sugar rings. The spectra with the CSA filter and dipolar dephasing of wheat straw samples, whether under anaerobic or aerobic conditions, showed very small nonprotonated quaternary carbon (OCqO) signals above the baselines, whereas those after the CSA filter and short CP displayed significant OCHO bands, similar to the OCO bands in the CSA-filtered spectra, revealing that OCO groups are dominantly protonated in all the samples. Compared with the CSA-filtered spectra of the 3-month decomposing wheat straw under anaerobic conditions, smaller peaks for O–C–O (around 105 ppm) and OCH (near 72 ppm) were observed in the 3-month decomposing wheat straw under aerobic conditions, suggesting faster loss of carbohydrates from wheat straw under aerobic conditions.

The spectra with only CH_n_ groups can be found in Fig 5k and 5l. These spectra for the 3-month decomposing wheat straw under anaerobic and aerobic conditions were similar, showing signals of protonated aromatics between 113 and 142 ppm, OCHO around 105 ppm, OCH centered around 72 ppm, OCH_2_ around 64 ppm, NCH between 50 and 60 ppm, CCH_2_C around 30 ppm, and CCH_3_ around 22 ppm. However, bands between 50 and 60 ppm for the decomposing wheat straw under anaerobic conditions were broader than those under the aerobic conditions, suggesting more NCH and thus more proteins or peptides.

The differences in the relative abundance of functional groups that are interpreted from the ^13^C multiCP NMR spectra in decomposing wheat straw residues under anaerobic and aerobic conditions are summarized in Table 3. The main functional group of wheat straw in the multiCP spectra (Fig 4 thin line) was O-alkyl C (64–93 ppm), which accounted for 54.2% of the C in the initial wheat straw prior to incubation, and then gradually decreased to 44.1% and 47.1% in decomposed residues under anaerobic and aerobic conditions, respectively, after incubation for 12 months. The percentage of NCH (44–64ppm) in decomposing wheat straw residue increased from 9.9% to 11.9%, and then to 13.1% under anaerobic conditions, while under aerobic conditions it increased from 9.9% to 11.4% during the first month, and then gradually declined to 8.6% by the end of 12 months.

**Table 3:** Temporal change of relative abundances of functional groups (%) in wheat straw obtained by multiCP NMR technique under anaerobic and aerobic conditions.

Incubation	0–44 ppm		44–64 ppm				64–93 ppm		93–113 ppm		113–142 ppm		142–162 ppm		162–188 ppm		188–220 ppm	
Time	Alkyl C		OCH_3_		NCH		O-alkyl C		Anomeric C		Aromatic C-C		Aromatic C-O		COO/N-C = O		C = O	
(months)	Ana	Aer	Ana	Aer	Ana	Aer	Ana	Aer	Ana	Aer	Ana	Aer	Ana	Aer	Ana	Aer	Ana	Aer
0	12.4	12.4	4.1	4.1	9.9	9.9	54.2	54.2	13.3	13.3	3.9	3.9	1.0	1.0	1.0	1.0	0	0
1	11.8	9.6	3.2	3.1	11.9	11.4	51.6	54.3	13.4	14.1	4.8	4.7	2.0	2.2	2.0	2.2	0	0
3	19.8	19.6	3.4	3.9	12.2	7.4	43.1	39.0	11.3	10.8	6.0	7.4	2.6	2.9	2.6	2.9	0	0
6	13.0	10.9	4.0	4.0	11.9	9.2	46.1	45.1	12.7	13.4	7.0	9.0	3.3	4.0	3.3	4.0	0	0.8
12	14.6	8.3	4.3	4.0	13.1	8.6	44.1	47.1	12.3	13.7	7.0	8.9	3.2	4.6	3.2	4.6	0	0.9

Note: Ana—anaerobic conditions; Aer—aerobic conditions.

The relative abundance of alkyl C (0–44 ppm) decreased in the first 1 month, gradually increased from month 1 to month 3, and finally decreased from 3 to 12 months under both anaerobic and aerobic conditions. The relative abundance of aromatic C (113–142 ppm), aromatic C-O (142–162 ppm), and COO/N-C = O (162–188 ppm) all increased under both anaerobic and aerobic conditions over the entire incubation period. After 12 months of decomposition, wheat straw residues showed an increase of the relative abundances of alkyl C, aromatic C, aromatic C-O, and COO/N-C = O but a decrease of the relative abundance of O-alkyl C compared to that of the original wheat residues. The aromatic C-O reflected characteristic of lignin and its residues (lignin thereafter), demonstrating that the decomposing wheat straw was more enriched in lignin than was the original straw, likely through selective preservation.

The decomposition of wheat straw under anaerobic conditions was slower than under aerobic conditions. After 6 and 12 months of decomposition, the relative abundances of O-alkyl C, NCH and alkyl C in decomposed wheat straw residues under anaerobic conditions were higher than those under aerobic conditions, while the relative abundances of the aromatic C, aromatic C-O and COO/N-C = O in decomposed wheat straw residues under anaerobic conditions were lower than those under aerobic conditions.

### Decomposition indices

**Fig 6:**
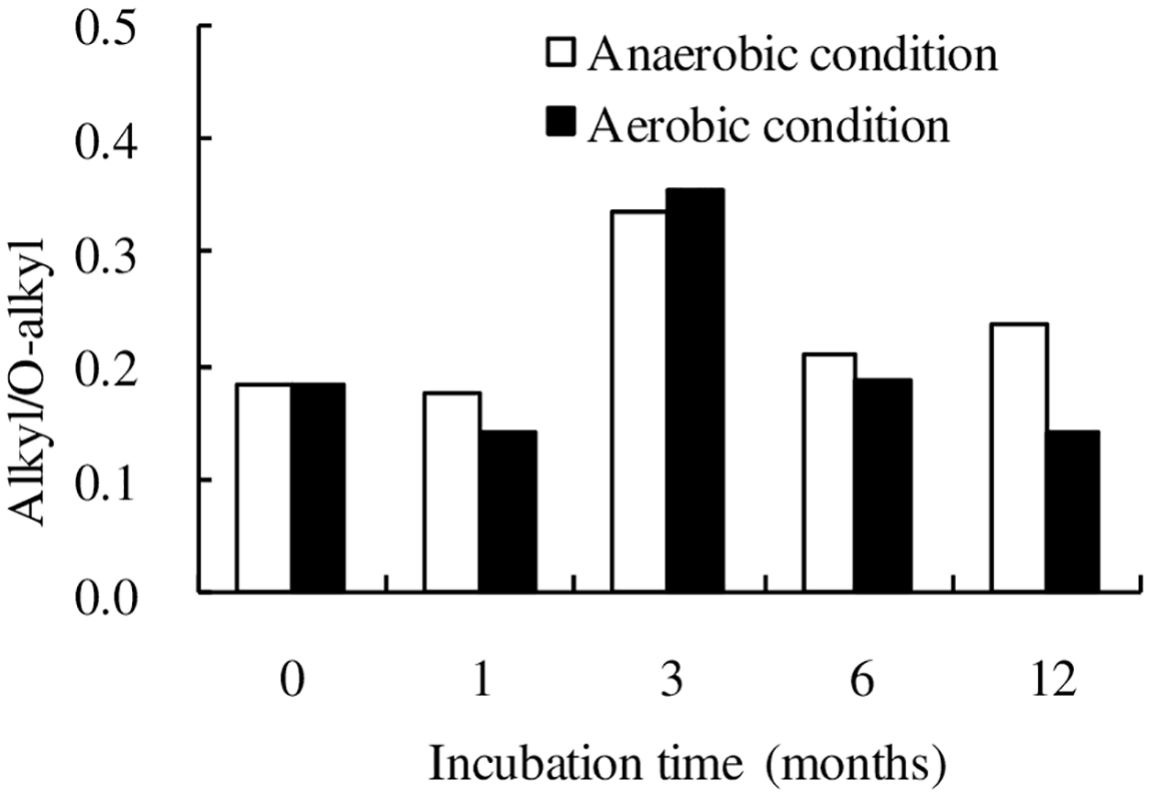
Temporal change of alkyl/O-alkyl ratio of wheat straw under anaerobic and aerobic conditions during decomposition. Alkyl refers to the multi CP NMR spectra region at 0–44 ppm, O-alkyl refers to the spectra region at 44–93 ppm.

The ratio of alkyl/O-alkyl (A/OA) has been used as an index of decomposition dynamics (Fig 6) [46]. The A/OA ratios of wheat straw residue declined slightly in the first month under aerobic conditions, rapidly increased from 1 month to 3 months, and then decreased gradually from 3 months to 12 months under both anaerobic and aerobic conditions. The A/OA ratios of wheat straw residues under anaerobic conditions were higher than those under aerobic conditions in the 1^th^, 6^th^ and 12^th^ months of incubation, but it was lower than under aerobic conditions after 3 months of incubation.

## Discussion

### Loss of mass, C and N in the wheat straw residues

The trend of mass loss of wheat straw is similar to those of previous studies on crop straw and other plant litters [11, 47]. Decomposition rates of crop straw were higher in the initial months, followed by lower decomposition rates in the remaining incubation stages [48, 9]. In the present study, most of the wheat straw mass was lost during the first half year, which accounted for 54.8% and 58.7% of its original mass. This is in agreement with the results from wheat and barley decomposition trials [24, 48], and likely due to the release of CO_2_ into ambient enviroment, or the release of relatively water-soluble substances such as pectin, free amino acids, peptides, organic acids as well as the mineral elements from the plant residue into the soil solution [14]. In this study, about 50% of the carbon and nitrogen, as well as more than 90% of hemicellulose, were lost from wheat straw at the beginning of the incubation. The soluble substances released into soil provided sufficient nutrients and energy for further microbial growth and decomposition of the crop residues. The slowly degradable plant components, such as lignin, accumulated in the crop residues [11, 45]. The remaining soluble substances in the plant residues declined to relatively low levels, and thus limited microbial growth and reduced the decomposition rate of plant residues [14]. Plant residues with high lignin-to-carbohydrate ratios may decompose more slowly than residues with low lignin-to-carbohydrate ratios, and high lignin to nitrogen ratios have also been correlated with slower decomposition rates [9]. Our results indicate that the decomposition rates declined as the ratios of lignin-to-carbohydrate increased because of the loss of cellulose and hemicellulose. Previous studies have also found that decomposition of crop residues promotes accumulation of lignin residues in soils, consistent with the fact that crop residues are likely one of the main parent materials of new SOM [23].

### Chemical structure alterations during decomposition of wheat straw residues

The main alterations in chemical structures during the decomposition of wheat straw were a decrease in the relative abundances of O-alkyl C and an increase in the relative abundances of alkyl C, aromatic C, aromatic C-O and COO/N-C = O functional groups. This result is consistent with several previous findings that a decrease of O-alkyl C and an increase of alkyl C during the decomposition of peats [49], forest litter [46] and crop straw [50]. Alkyl C (0–44 ppm) intensity mainly comes from plant waxes, cutin and suberin[51]. An increase in alkyl C during the decomposition process has been attributed to an increase in cross-linking of long chain alkyl compounds or to the selective preservation of resistant or insoluble, aliphatic macromolecules [52]. Preston reported that an accumulation of alkyl C signals was also associated with the loss of the most easily metabolizable molecules [45].

The decrease in O-alkyl C (the 64–93 ppm NMR signal) in the wheat straw residues is ascribed to the decline of polysaccharides such as cellulose and hemicelluloses [35], and it is consistent with the observed decline in cellulose and hemicellulose concentrations. The increase of the relative abundances of aromatic C (113–142 ppm) and aromatic C-O could be attributed to the slower degradation and thus selective preservation of lignin or partially degraded lignin structures [52]. These results were confirmed by the consistent increase in relative mass percentage of lignin in wheat straw residues during the process of decomposition (Fig 3). The increase in the relative abundances of the COO/N-C = O (142–162 ppm) functional groups could be explained by loss of the easily degradable carbohydrates in wheat straw residues. These interpretations are derived from changes in the ^13^C multiCP and multiCP /DD spectra (Figs 4 and 5). The results confirm our hypothesis that the chemical composition of wheat straw residues changed during decomposition under both anaerobic and aerobic conditions, potentially affecting the structure and composition of SOM and the availability of soil nutrients.

### Decomposition of wheat straw residues in response to soil aeration

Soil oxidation-reduction potential in anaerobic conditions was significantly different from that in the aerobic condition, which influenced the active microbial species and thus impacted litter decomposition [53]. In the present study, oxidation-reduction potentials between -150 and -50 mv, and between 150 and 250 mv were maintained under anaerobic and aerobic conditions, respectively, by flooding and maintaining 75%~80% of the maximum water-holding capacity of soil by irrigation. The remaining mass percentage of lignin in wheat straw residues gradually increased (from 10.1% to 21.8%) under anaerobic conditions as the carbohydrates, including cellulose and hemicellulose, were degraded by bacterial decomposers during the entire 12-month incubation (Fig 3). In flooded cropping systems, lignin residues may accumulate in plant litter residues and soil because of high input rates through annual crop straw incorporation, as well as their slower decomposition under anaerobic conditions [22, 20]. For example, soil humic acid contained more lignin derivatives in a triple-cropped irrigated lowland (anaerobic) rice soil than that in a nearby single-cropped aerobic rice soil [22]. This may be attributed to the lack of soil oxygen that would limit the activity of fungi and reduce the production of lignases [22]. The mass percentage of lignin remaining in decomposing wheat straw residues under anaerobic conditions was higher by 10.5% to 20.5% than under aerobic conditions in the 12-month incubation period (Fig 3). Results from multiCP spectra also showed that the signals of aromatic C and alkyl C in the decomposing wheat straw residues at 12 months under anaerobic condition were higher than under aerobic conditions (Fig 4). This is in line with our hypothesis that the concentrations of C-containing functional groups in decomposing wheat residues may vary depending on anaerobic and aerobic conditions. The higher percentage of recalcitrant molecules, such as lignin, in decomposing residues under anaerobic condition could be attributed to the slower degradation and thus accumulation of lignin or partially degraded lignin structures in decomposed residues under anaerobic condition [22]. Lignin residues in wheat straw decomposing under anaerobic conditions may be precursors to some SOM components such as mobile humic acid (MHA) or calcium-bound humic acid (CaHA), a fraction that has been hypothesized to immobilize labile soil nitrogen and reduce nitrogen availability [23].

## Conclusions

Our study illustrates that soil anaerobic and aerobic conditions significantly affected the chemical structure of wheat straw residues during 12 months of decomposition. We combined the semi-quantitative assessment of cell wall components including lignin, hemicelluloses and cellulose with quantitative NMR measurements, and independent and semi-quantitative measurements of decomposition rates. The higher mass percentages of lignin and the higher signals of aromatic C and alkyl C functional groups in residues decomposing under anaerobic conditions compared with those under aerobic conditions were consistent with the slower decomposition rates of aryl C and alkyl C. These differences might be related to the dynamics of nitrogen mineralization and labile nitrogen forms in soil. However, further studies are needed to examine the quantitative differences in the chemical structures of soil organic matter between anaerobic and aerobic conditions, as well as in the N forms and availability.
